# Association of sun-seeking behaviors with indoor tanning habit in US females

**DOI:** 10.21203/rs.3.rs-2899336/v1

**Published:** 2023-05-23

**Authors:** Bojung Seo, Sheng Yang, Eunyoung Cho, Abrar A Qureshi, Jiali Han

**Affiliations:** Indiana University; Indiana University; Alpert Medical School of Brown University; Alpert Medical School of Brown University; Indiana University

## Abstract

**Background:**

Frequent exposure to ultraviolet light in early life has more detrimental and long-term effects on skin than in adulthood. Teenagers with strong sun-seeking behaviors may be more likely to use an indoor tanning bed than those who seek less sun, probably due to addictiveness of ultraviolet exposure. We aimed to examine associations between sun exposure behaviors and average annual indoor tanning usage frequency during high school/college in US females.

**Methods:**

In this cross-sectional study, we used data from The Nurses’ Health Study II, a large prospective cohort of US female nurses. We included a total of 81,746 white females who answered the average annual frequency of indoor tanning during high school/college. Our study exposures were average weekly time spent outdoors in a swimsuit and average percentage of time of wearing sunscreen at the pool or beach during their teenage years, average weekly hours spent outdoors in direct sunlight in daytime during high school/college, and the number of severe sunburns which blistered between ages 15–20. Main outcomes was average annual frequency of indoor tanning bed usage during high school/college.

**Results:**

In multivariable-adjusted logistic regression, we demonstrated positive associations between the sun exposure behaviors and the indoor tanning habit. Specifically, teenagers who spent daily outdoors in a swimsuit (adjusted odds ratio [aOR], 95% confidence interval [CI] for daily vs. <1/week: 2.68, 1.76–4.09) or who had ≥ 10 sunburns that blistered (aOR, 95% CI for ≥ 10 vs. never: 2.18, 1.53–3.10) were more likely to use indoor tanning beds ≥ 12 times/year. Also, teenagers/undergraduates who spent ≥ 5hours/week outdoors in direct sunlight during daytime used indoor tanning ≥ 12 times/year (aOR, 95% CI: 2.18, 1.39–3.44) than those who spent < 1/week. However, there was not a significant association between average uses of sunscreen at the pool/beach and indoor tanning bed. Multivariable-adjusted linear regression models also showed similar results.

**Conclusions:**

Teenagers who spent more time outdoors or got more sunburns tended to use indoor tanning more frequently. These findings provide evidence that teenagers with strong sun-seeking behaviors may have excessive exposure to artificial ultraviolet radiation as well.

## Introduction

Skin cancer is the most common malignancy in the United States (1). Ultraviolet (UV) radiation is the major etiologic agent of skin cancer development and has been classified as “carcinogenic to humans” (group1) by the International Agency for Research on Cancer in 2009 (2). Tanning is defined as intentional exposure to UV radiation that darkens the skin for cosmetic purposes, typically achieved through outdoor sun exposure for a long time or use of indoor tanning beds that emit artificial UV radiation (3, 4). Despite risks associated with tanning, people still pursue it, probably due to the reinforcing effects of UV radiation on tanning behaviors. Such reinforcing effects are created by the many neuropeptides released from the skin after exposure to UV radiation, which can offer relaxation and increase the sense of well-being (5, 6). In fact, tanning is becoming more and more popular in the US. Among American college students, over 70% students intentionally seek a tan outdoors (7); according to a review published in 2018 (3), more than 10 million people use indoor tanning each year in the US, many of whom are adolescent girls and young females (8). However, exposure to UV in early ages has more harmful and longer effects on skin cancer risk than in adults (9). Therefore, understanding tanning behaviors during early life is important for skin cancer prevention and reduction in burden of the skin cancer among young US females.

Among 6,903 non-Hispanic white adolescents aged 13 to 19 years (51.4% females), frequent sunbathers were females and far more likely to use indoor tanning beds than those who never sunbathed (10). Similarly, another study consisting of 281 white females under age 40 reported that female indoor tanners (73.3% of the females in the sample) during early ages more frequently sunbathed than females who never tanned indoors (11). However, these studies were limited by a lack of information of frequency of indoor tanning bed use during early life and outdoor sun-seeking behaviors as well as modest sample size. In addition, the studies reported that females excessively used tanning beds more than males during early life indicating that females are more likely to develop skin cancer and benefit from skin cancer screening (10, 11). Accordingly, female-focused larger investigation on association between outdoor and indoor tanning behaviors in their early ages is warranted.

Given the above facts, we hypothesized that females who had stronger sun-seeking behaviors would tend to use indoor tanning facilities more frequently. Thus, we sought to investigate the association between a series of outdoor sun-seeking behaviors and frequency of indoor tanning using data from the Nurses’ Health Study II (NHS II), a large well-characterized cohort of US females.

## Methods

### Study Population

The NHS II is a large prospective cohort focusing on health and disease risk factors. In 1989, the cohort enrolled 116,429 female nurses aged 25 to 42 years who resided in 1 of 14 US states with a large number of registered nurses. The initial self-administered questionnaires asked demographic factors, medical/familial histories, and health-related information of their early life. Some health habits in early ages were additionally asked in follow-up surveys. Details of this cohort have been described previously (12). In this study, we included white females who answered the average annual frequency of indoor tanning during high school/college. The study protocol was approved by the institutional review boards of the Brigham and Women’s Hospital and Harvard T.H. Chan School of Public Health, and those of participating registries as required. Written informed consent was also obtained from all study participants. This study followed the Strengthening the Reporting of Observational Studies in Epidemiology (STROBE) reporting guidelines.

### Outcome

Participants reported their frequency of tanning bed use during high school/college by choosing from the following 6 categories: none, 1–2, 3–5, 6–11, 12–23, and ≥ 24 times per year in questionnaire. We recategorized the average annual indoor tanning frequencies to 4 categories (none, 1–2, 3–11, and ≥ 12 times per year). Also, we converted the responses from the original 6 categories to continuous values by assuming each level of the categories as 0, 1.5, 4.5, 9, 18, and 24 times per year, respectively (13).

### Exposures

We collected participants’ responses to the outdoor sun-seeking behavior-related questions that include average weekly time spent outdoors in a swimming suit as a teenager (5 categories: <1, 1, 2, several, and daily per week), average percentage of time of wearing sunscreen at the pool/beach as a teenager (5 categories: 100%, 75%, 50%, 25%, and 0%), average weekly hours spent outdoors in direct sunlight in daytime during high school/college (3 categories: <1, 2–4, and ≥ 5 hours per week), and the number of severe sunburns which blistered between ages 15–20 (5 categories: never, 1–2, 3–4, 5–9, and ≥ 10 times).

### Covariates

We included the following participants-reported variables as our covariates - age, average number of cigarettes per day at age 15–24, number of drinks of alcohol at age 15–22, average frequencies of strenuous physical activity/sports at least twice per week during high school and ages 18–22, hair color (red, blonde, light brown, dark brown and black), family history of melanoma (yes vs. no), personal history of major chronic diseases (yes vs. no), and moles on lower legs (none, 1–2, 3–9, and ≥ 10). We defined a participant had major chronic diseases when they reported any cancer, myocardial infarction, stroke, type 2 diabetes, hypertension, inflammatory bowel disease, rheumatoid arthritis, and/or multiple sclerosis in their medical history question.

### Statistical Analysis

We calculated age-standardized characteristics and outdoor sun-seeking behaviors according to average annual frequency of indoor tanning bed usage during high school/college. Continuous variables were presented as mean (standard deviation, SD) and categorical variables were presented as percentage. We performed age and multivariable-adjusted multinomial logistic regression using the 4 categorized outcome to calculate adjusted odds ratios (aORs) with 95% confidence intervals (CIs) for the association between outdoor sun-seeking behaviors and the average annual frequency of indoor tanning bed use during early life. In the multivariable-adjusted model, for each exposure variable, we adjusted for all covariates described above. In the same way, we conducted age- and multivariable-adjusted linear regression but using the continuous outcome values, yielding adjusted *β* (95% CI). In all regression models, to reduce the number of observations deleted because of missing values in covariates, we created indicator variables of the missing data in all covariates and adjusted them as well.

All statistical tests were two-sided, and we determined statistical significance using a threshold of p-value < 0.05. All analyses were carried out using Statistical Analysis System software (version 9.4; SAS Institute, Cary, NC).

## Results

Age-standardized characteristics of participants according to frequencies of tanning bed usage during high school/college are shown in Table 1. A total of 81,746 eligible female nurses were included in the study. 7,415 (9.1%) females used indoor tanning beds during high school/college, among whom 1,227 (1.5%) reported that they used indoor tanning beds with high frequency (≥ 12 times/year). Females who used tanning beds more often during early life tended to be younger and were heavier smokers and drinkers during their early life. They also tended to engage in more strenuous physical activity or sports during their early life and had higher nevus counts on their lower legs. The distributions of other characteristics, such as hair color, family history of melanoma, and history of major chronic diseases, were similar across the 4 categories of the average frequency of indoor tanning bed usage.

The age- and multivariable-adjusted odds ratios for the association between outdoor sun-seeking behaviors and indoor tanning frequency during high school or college are presented in Table 2. In multivariable-adjusted models, being outdoors more often in a swimsuit as a teenager, spending more time outdoors in direct sunlight in the middle of the day during high school/college, and experiencing more severe sunburns between ages 15–20 were significantly associated with higher frequency of average annual indoor tanning bed usage. Specifically, teenagers who spent daily outdoors in a swimsuit (aOR, 95% CI for daily vs. <1 per week: 2.68, 1.76–4.09) or who had ≥ 10 sunburns that blistered (aOR, 95% CI for ≥ 10 vs. never: 2.18, 1.53–3.10) were more likely to use indoor tanning beds ≥ 12 times/year. Also, teenagers/undergraduates who spent ≥ 5 hours per week outdoors in direct sunlight during daytime used indoor tanning ≥ 12 times/year (aOR, 95% CI: 2.18, 1.39–3.44) than those who spent < 1 per week. However, there was not a significant association between sunscreen usage at the pool/beach and the frequency of indoor tanning bed usage.

Table 3 shows the linear regression results on the association. Overall, the significant associations we observed in the logistic regression were also seen in the linear models. Teenagers who spent daily outdoors in a swimsuit (adjusted *β*, 95% CI for daily vs. <1 per week: 0.25, 0.16–0.34) or who had ≥ 10 sunburns that blistered (adjusted *β*, 95% CI for ≥ 10 vs. never: 0.45, 0.29–0.62) were more likely to use indoor tanning beds. Also, teenagers/undergraduates who spent ≥ 5 hours per week outdoors in direct sunlight during daytime used indoor tanning more (adjusted *β*, 95% CI: 0.29, 0.18–0.40) than those who spent < 1 per week.

## Discussion

Here we investigated the association between outdoor sun-seeking behaviors and average annual frequency of indoor tanning during early life using the observational data from the NHS II. We found that females who spent more time outdoors in a swimsuit or in direct sunlight in the middle of the day, or experienced more severe sunburns were higher-frequency indoor tanners during their early life.

The significant association between outdoor sun-seeking behaviors and indoor tanning habit should cause concern, because exposure to excessive UV radiation from indoor tanning and spending outdoor in direct natural sunlight can be made (10) and such excessive exposure leads to profound health risks, *e.g*., atrophy, pigmentary changes, wrinkling, and malignancy (14). Furthermore, It is well-known that such exposure is linked to three most common types of skin cancer, *i.e*., basal cell carcinoma, squamous cell carcinoma, and malignant melanoma (15), among which melanoma is the leading cause of death in US females aged 25 to 30 and second leading cause of death in females aged 30 to 35 (16). Despite the risks, why the outdoor sun-seeking behaviors and indoor tanning habit were significantly associated can be explained by the known fact that UV radiation can increase the blood flow associated with central reward mechanisms to encourage excessive tanning (17). To be specific, exposure to UV radiation, triggering DNA damage to the skin, activates the p53 protein to induce production of pro-opiomelanocortin (POMC) and its derivative *β*-endorphin (18). *β*-endorphin, the most abundant endogenous opioid, acts on central neural dopamine receptors to make the tanner relaxed and increase her sense of well-being (5, 19). Moreover, frequent tanning may chronically elevate tanners’ endorphin levels, creating reinforcing effects (20).

We observed significant associations between the time spent outdoors in a swimsuit or in direct sunlight in daytime as a teenager and indoor tanning bed use during high school/college. Weekly frequency of spending outdoors wearing a swimsuit or in direct sunlight reflect almost the same characteristics of a participant, *i.e*., an individual’s sun-seeking degree and both in high frequency may come with higher risk for skin cancer because of the increased exposure to UV rays. This can also strengthen stimulation of the reward system and increase the sun-seeking desire more (21). Such increased sun-seeking needs may also lead to increased use of indoor tanning facilities that emits far more intense UV lights than that of natural sunlight, which in turn becomes a series of vicious cycles (17). Thus, education should be provided to teenagers with strong sun-seeking behaviors to prevent them from being long exposed to direct sunlight outside in the middle of the day or to artificial UV lights in indoor equipment. Furthermore, because teenagers’ behavior is more likely to be influenced by others because of the psychological pressure to conform (22, 23), school health stakeholders or parents should make teenagers keep practicing sun safety according to their own will.

Sunburn is an inflammatory reaction to UV radiation damage to skin’s outermost layers (24). Repeated sunburns raise the risk for skin cancer by an altered tumor-suppressing gene that lowers the chance to prevent from progressing to cancer (25). Especially, blistering sunburns that occur in childhood or adolescence pose the greatest risk for cutaneous melanoma in adulthood (26). Five or more sunburns increased the risk of development of melanoma (27). In addition to the severe sunburns, indoor tanning bed use is also an established risk factor for melanoma (28, 29). In our study, we found the strongest positive associations between undergoing ≥ 10 severe sunburns which blistered between ages 15–20 and usage of indoor tanning beds of ≥ 12. Such relationship was also observed in other studies reporting that indoor tanning was positively associated with sunburns because of their tan-seeking behaviors (30, 31). To mitigate the aggravated risk, dermatologists may need to make a consensus on the proper guideline of indoor tanning usage in teenagers with severe sunburns and public health offi cers or school health teachers also need to provide preventive education for protecting teenagers from severe sunburns, such as encouragement of use of sunscreen outdoors.

Sunscreens are an important protection against UV exposure and can effectively reduce the incidence of skin cancers (32). However, our study did not demonstrate the inverse association between the percentage of time of wearing sunscreen at the pool/beach and the use of indoor tanning beds, which is a different result from previous findings (22, 33, 34). A study among US high school students found that white female students who reported always using sunscreen were significantly less likely to use indoor tanning equipment (33). Another study pointed out that non-use of sunscreen at the pool/beach was an independent predictor of indoor tanning use habit (22). Moreover, a study based on US 2015 National Health Interview Survey data also found that those who frequently tanned indoors were more likely to rarely/never use sunscreen (34). It might be due to safety frigidity that frequent users of indoor tanning beds were less likely to wear sunscreen, even though sunscreen use is the one of most important aspects of sun protective behavior. In addition, they might think using sunscreen would prevent the bronzing of their skin especially in the 70s and 80s when there were not bronzing sunscreens. In fact, inconvenience and no perceived need for applying sunscreen are known as the major reasons for not using sunscreen (35). However, the indoor tanning-seeking behavior may be more likely to be due to addictiveness of UV exposure regardless of how they perceive the importance of the use of sunscreen, which may partially explain such no association we observed in our study.

Many countries have recently moved to ban those younger than 18 from using indoor tanning beds (36–38). However, adults who use tanning beds also deserve special attention, especially those under age 25. Our data showed that people who used tanning beds more often during early life tended to be younger, and also had more moles on their lower legs, which was consistent with the results of a previous study (13). As shown in Table 1, heavier smokers as well as drinkers tended to use tanning beds more often. As the reward mechanisms affected by smoking are similar to those associated with indoor tanning bed use (39), those interactions deserve further study. Females who engaged in more strenuous physical activity or sports sought to use indoor tanning beds more, which might be due to needs for relaxing and relieving pain. Persistent indoor tanning is positively associated with both risk taking (40) and unhealthy lifestyle (10) behaviors. Therefore, those results suggest that teenagers with a cluster of such unhealthy behaviors should be paid closer attention by considering them as high-risk groups.

Our study has several strengths, including a relatively large sample size (N = 81,746) compared to the previous studies with similar research topics. In addition, detailed collection of baseline, lifestyle, and medical history using well-designed and validated self-reported questionnaires allowed us to adjust for widely recognized potential confounders in the relationship of interest and to develop valid reference for the association. Moreover, we used the average data of high-quality sun-seeking or indoor tanning-seeking behaviors during high school/college, which may be more valuable than data or records during shorter period in measuring habit of the participants that affected their indoor tanning facilities usage during early life.

Our study also has several limitations. First, the information we collected on study variables may introduce recall bias; however, there was only slim chance that participants would exaggerate or understate their actual habit/values because when they reported, they did not have any pressure to do so in cohort study design. Second, residual and unmeasured confounding cannot be fully ruled out because of the nature of the observational data; but we adjusted many well-known confounding factors. Third, some misclassification is inherent from self-reported questionnaires; yet the questionnaire has been extensively validated in subsamples of this cohort, and any misclassification would likely be some nondifferential error to bias our results toward the null. Lastly, we studied only compliant healthy professional female nurses belonging to a specific social stratum, which may create a lack of external validity; nevertheless, the results could be applied to females with similar sun-seeking behaviors and lifestyles.

In conclusion, our study reveals associations between outdoor sun-seeking behaviors and average annual frequency of indoor tanning bed usage, suggesting that young females who spent more time outdoors and had more severe sunburns tended to use indoor tanning more frequently than those with fewer sun-seeking behaviors. If properly publicized, these findings could change perceptions about indoor tanning bed usage and guide potential interventions among young females with strong outdoor sun-seeking behaviors. This work may contribute vital information for public health messaging about young female skin health and guide potential interventions for control of sun-seeking behaviors and indoor tanning use in high-risk groups.

## Figures and Tables

**Table 1:** Age-standardized characteristics according to average annual frequency of indoor tanning bed usage during high school/college in US females^a^

Characteristics	None	1–2	3–11	≥12
**Number of participants**	74,331	3,400	2,788	1,227
**Age, years**	34.8 (4.6)	32.8 (5.0)	32.2 (5.0)	31.3 (4.8)
**Average number of cigarettes per day at age 15–24**	11.1 (7.6)	10.9 (8.1)	11.1 (8.4)	12.1 (8.6)
**Number of drinks of alcohol at age 15–22** ^b^	1.8 (2.9)	2.1 (2.9)	2.3 (3.2)	2.5 (3.5)
**Average frequencies of strenuous physical activity/sports at least twice per week during high school and ages 18–22** ^c^	4.7 (3.6)	5.0 (3.6)	5.1 (3.6)	5.2 (3.6)
**Hair color**				
Red, %	3.6	2.7	3.5	1.9
Blonde, %	14.6	16.1	16.1	17.8
Dark brown, %	35.7	34.1	36.2	32.8
Black, %	3.1	1.2	1.3	0.8
**Family history of melanoma, %**	4.2	4.8	6.5	5.0
**History of major chronic diseases, %** ^d^	12.3	12.7	10.9	10.6
**The number of moles on lower legs**				
1–2, %	18.5	18.4	19.6	18.0
3–9, %	16.6	17.3	18.4	17.0
10+, %	14.1	16.9	15.2	19.7
**Average weekly time spent outdoors in a swimsuit as a teenager**				
1, %	9.5	8.3	7.3	4.8
2, %	16.0	14.9	14.0	10.6
Several, %	45.1	50.9	51.7	53.9
Daily, %	15.4	18.0	20.4	25.1
**Average percentage of time of wearing sunscreen at the pool or beach as a teenager**				
75, %	4.1	3.7	3.1	3.1
50, %	9.9	9.7	9.4	7.9
25, %	22.4	26.2	23.7	20.5
0, %	62.2	59.5	63.1	67.0
**Average weekly hours spent outdoors in direct sunlight in the middle of the day during high school/college**				
2–4, %	32.9	31.1	24.0	19.6
5+, %	59.6	64.2	72.8	77.2
**The number of severe sunburns which blistered between ages 15–20**				
1–2, %	38.3	42.6	40.4	39.0
3–4, %	16.6	20.2	18.4	18.3
5–9, %	7.2	8.6	9.7	11.0
10+, %	2.4	3.1	3.6	5.6

Note:

a) Values are means (SD) for continuous variables, percentages for categorical variables, and are standardized to the age distribution of the study population except for baseline age;

b) Number of usual drinks equals total of bottles/cans of beer, plus 4 oz. glasses of wine, plus shots of liquor;

c) Strenuous (aerobic) physical activity or sports mean swimming, aerobics, field hockey, basketball, cycling, and running;

d) Major chronic diseases include cancer, myocardial infarction, stroke, type 2 diabetes, hypertension, inflammatory bowel disease, rheumatoid arthritis, and multiple sclerosis.

**Table 2:** Age and multivariable-adjusted logistic regression for the association of sun-seeking behaviors with average annual frequency of indoor tanning bed usage during high school/college

	None	1–2			3–11			≥12		
	N^a^	N^a^	Model 1	Model 2^b^	N^a^	Model 1	Model 2^b^	N^a^	Model 1	Model 2^b^
**Average weekly time spent outdoors in a swimsuit as a teenager**										
<1	9,270	215	1.00	1.00	140	1.00	1.00	54	1.00	1.00
1	6,323	230	1.49 (1.23–1.79)	1.31 (1.00–1.71)	164	1.60 (1.27–2.01)	1.45 (1.06–1.98)	51	1.25 (0.85–1.84)	1.34 (0.79–2.27)
2	10,603	444	1.65 (1.40–1.95)	1.50 (1.19–1.89)	331	1.84 (1.51–2.25)	1.44 (1.09–1.91)	110	1.52 (1.10–2.11)	1.22 (0.76–1.96)
Several	29,759	1,553	1.99 (1.72–2.30)	1.69 (1.38–2.07)	1,304	2.48 (2.08–2.96)	2.01 (1.58–2.55)	578	2.70 (2.04–3.58)	2.45 (1.65–3.64)
Daily	10,133	559	2.07 (1.77–2.44)	1.80 (1.43–2.26)	523	2.87 (2.37–3.47)	2.25 (1.73–2.93)	276	3.71 (2.76–4.98)	2.68 (1.76–4.09)
**Average percentage of time of wearing sunscreen at the pool or beach as a teenager**										
100%	950	34	1.00	1.00	20	1.00	1.00	18	1.00	1.00
75%	2,575	127	1.29 (0.88–1.90)	1.16 (0.68–1.96)	92	1.56 (0.95–2.55)	2.23 (1.05–4.76)	44	0.81 (0.46–1.41)	0.63 (0.29–1.38)
50%	6,296	317	1.34 (0.93–1.92)	1.09 (0.67–1.78)	270	1.91 (1.20–3.02)	2.39 (1.16–4.92)	101	0.78 (0.47–1.30)	0.66 (0.33–1.33)
25%	14,310	871	1.67 (1.18–2.37)	1.42 (0.89–2.27)	660	2.14 (1.37–3.37)	2.43 (1.19–4.95)	266	0.96 (0.59–1.56)	0.88 (0.46–1.70)
0%	40,614	1,646	1.31 (0.93–1.85)	1.07 (0.67–1.71)	1,412	2.03 (1.30–3.17)	2.29 (1.13–4.64)	635	1.11 (0.69–1.78)	0.88 (0.46–1.69)
**Average weekly hours spent outdoors in direct sunlight in the middle of the day during high school/college**										
**Average weekly time spent outdoors in a swimsuit as a teenager**										
<1	5,574	142	1.00	1.00	76	1.00	1.00	33	1.00	1.00
2–4	24,162	1,013	1.53 (1.28–1.83)	1.59 (1.23–2.06)	655	1.80 (1.42–2.29)	1.86 (1.31–2.65)	222	1.36 (0.94–1.96)	1.12 (0.70–1.81)
≥5	43,564	2,221	1.76 (1.48–2.09)	1.59 (1.24–2.04)	2,033	2.89 (2.29–3.64)	2.83 (2.01–3.98)	958	2.96 (2.09–4.20)	2.18 (1.39–3.44)
**The number of severe sunburns which blistered between ages 15–20**										
Never	26,144	911	1.00	1.00	784	1.00	1.00	341	1.00	1.00
1–2	28,317	1,444	1.45 (1.33–1.58)	1.42 (1.25–1.62)	1,161	1.35 (1.23–1.48)	1.24 (1.08–1.42)	490	1.31 (1.14–1.50)	1.03 (0.84–1.26)
3–4	12,289	656	1.56 (1.41–1.73)	1.57 (1.35–1.82)	492	1.36 (1.22–1.53)	1.27 (1.08–1.51)	211	1.36 (1.14–1.61)	1.10 (0.87–1.41)
5–9	5,374	280	1.55 (1.35–1.77)	1.49 (1.22–1.81)	251	1.63 (1.41–1.88)	1.48 (1.20–1.82)	119	1.81 (1.46–2.23)	1.39 (1.04–1.87)
**Average weekly time spent outdoors in a swimsuit as a teenager**										
≥10	1,803	98	1.61 (1.30–2.00)	1.64 (1.24–2.15)	92	1.78 (1.43–2.23)	1.72 (1.30–2.29)	62	2.82 (2.14–3.72)	2.18 (1.53–3.10)

Note:

a) Number of participants;

b) Adjusted for age (continuous), average number of cigarettes per day at age 15–24 (continuous), number of drinks of alcohol at age 15–22 (continuous), average frequencies of strenuous physical activity/sports at least twice per week during high school and ages 18–22 (continuous), hair color (red, blonde, light brown, dark brown and black), family history of melanoma (yes, no), personal history of major chronic diseases (yes, no) and moles on lower legs (none, 1–2, 3–9, 10+).

**Table 3:** Age and multivariable-adjusted linear regression for the association of sun-seeking behaviors with average annual frequency of indoor tanning bed usage during high school/college

	N^a^	Model 1	Model 2^b^
**Average weekly time spent outdoors in a swimsuit as a teenager**			
<1	9,679	1.00	1.00
1	6,768	0.07 (−0.01–0.16)	0.05 (−0.07–0.17)
2	11,488	0.13 (0.05–0.20)	0.03 (−0.08–0.13)
Several	33,194	0.50 (0.42–0.57)	0.32 (0.21–0.43)
Daily	11,491	0.32 (0.26–0.38)	0.25 (0.16–0.34)
**Average percentage of time of wearing sunscreen at the pool or beach as a teenager**			
100%	1,022	1.00	1.00
75%	2,838	−0.002 (−0.20–0.19)	−0.07 (−0.35–0.21)
50%	6,984	0.03 (−0.15–0.21)	−0.02 (−0.28–0.23)
25%	16,107	0.12 (−0.05–0.30)	0.08 (−0.16–0.33)
0%	44,307	0.13 (−0.04–0.30)	0.03 (−0.21–0.27)
**Average weekly hours spent outdoors in direct sunlight in the middle of the day during high school/college**			
<1	5,825	1.00	1.00
2–4	26,052	0.09 (0.01–0.17)	0.07 (−0.04–0.18)
≥5	48,776	0.39 (0.31–0.46)	0.29 (0.18–0.40)
**The number of severe sunburns which blistered between ages 15–20**			
Never	28,180	1.00	1.00
1–2	31,412	0.13 (0.09–0.18)	0.01 (−0.05–0.08)
3–4	13,648	0.15 (0.10–0.21)	0.06 (−0.02–0.15)
5–9	6,024	0.29 (0.21–0.37)	0.17 (0.06–0.28)
≥10	2,055	0.53 (0.41–0.66)	0.45 (0.29–0.62)

Note:

a) Number of participants;

b) Adjusted for age (continuous), average number of cigarettes per day at age 15–24 (continuous), number of drinks of alcohol at age 15–22 (continuous), average frequencies of strenuous physical activity/sports at least twice per week during high school and ages 18–22 (continuous), hair color (red, blonde, light brown, dark brown and black), family history of melanoma (yes, no), personal history of major chronic diseases (yes, no) and moles on lower legs (none, 1–2, 3–9, 10+).

## Data Availability

The data that support the findings of this study are available from Brigham and Women’s Hospital and Harvard T.H. Chan School of Public Health but restrictions apply to the availability of these data, which were used under license for the current study, and so are not publicly available. Data described in the manuscript, code book, and analytic code are however available from the authors upon reasonable request to corresponding author and with permission of Brigham and Women’s Hospital and Harvard T.H. Chan School of Public Health.

## Key Points

### Question:

How are outdoor sun-seeking behaviors associated with indoor tanning frequency?

### Findings:

This large study using data from the Nurses’ Health Study II (NHSII) found that female who spent more time outdoors or got more sunburns tended to use indoor tanning more frequently than those who rarely or never engaged in outdoor sun-seeking behaviors.

### Meaning:

The findings reveal the associations between outdoor sun-seeking behaviors and frequency of indoor tanning, which could change perceptions about indoor tanning and guide potential interventions directed at young females with frequent outdoor sun-seeking behaviors.
